# Building a Strategic Framework for Comparative Effectiveness Research in Complementary and Integrative Medicine

**DOI:** 10.1155/2012/531096

**Published:** 2012-12-26

**Authors:** Claudia M. Witt, Margaret Chesney, Richard Gliklich, Lawrence Green, George Lewith, Bryan Luce, Anne McCaffrey, Shelly Rafferty Withers, Harold C. Sox, Sean Tunis, Brian M. Berman

**Affiliations:** ^1^Institute for Social Medicine, Epidemiology and Health Economics, Charité University Medical Center, Berlin, Germany; ^2^Center for Integrative Medicine, University of Maryland School of Medicine, Baltimore, MD, USA; ^3^Osher Center for Integrative Medicine, University of California, San Francisco, CA, USA; ^4^Quintiles Outcome, Durham, NC, USA; ^5^Department of Epidemiology and Biostatistics, University of California, San Francisco, CA, USA; ^6^Primary Care and Population Sciences, University of Southampton, Southampton, UK; ^7^Center for Health Economics and Science Policy, BioSource Corporation, Bethesda, MD, USA; ^8^Department of Pharmacy, University of Washington, Seattle, WA, USA; ^9^Marino Center for Integrative Health, Cambridge, MA, USA; ^10^The Institute for Integrative Health, Baltimore, MD, USA; ^11^Geisel School of Medicine, Dartmouth College, Hanover, NH, USA; ^12^Center for Medical Technology Policy, Baltimore, MD, USA

## Abstract

The increasing burden of chronic diseases presents not only challenges to the knowledge and expertise of the professional medical community, but also highlights the need to improve the quality and relevance of clinical research in this domain. Many patients now turn to complementary and integrative medicine (CIM) to treat their chronic illnesses; however, there is very little evidence to guide their decision-making in usual care. The following research recommendations were derived from a CIM Stakeholder Symposium on Comparative Effectiveness Research (CER): (1) CER studies should be made a priority in this field; (2) stakeholders should be engaged at every stage of the research; (3) CER study designs should highlight effectiveness over efficacy; (4) research questions should be well defined to enable the selection of an appropriate CER study design; (5) the CIM community should cultivate widely shared understandings, discourse, tools, and technologies to support the use and validity of CER methods; (6) Effectiveness Guidance Documents on methodological standards should be developed to shape future CER studies. CER is an emerging field and its development and impact must be reflected in future research strategies within CIM. This stakeholder symposium was a first step in providing systematic guidance for future CER in this field.

## 1. Introduction

The increasing burden of chronic diseases presents challenges not only to the knowledge and expertise of the professional medical community, but also to the socioeconomic stability of society. Chronic disease—which includes chronic pain, diabetes, and heart disease, among others—represents 75% of health care spending in the USA; patients with five or more chronic diseases account for 76% of Medicare spending [1]. 

Increasingly, treating patients' chronic illnesses requires complex interventions made up of various interconnecting parts [2]. Especially in a combination of interventions and the possible interactions between them, a more personalized treatment plan is called for. Complementary and integrative medicine is among the options that can be tailored for more personalized/individualized medicine. 

The National Center for Complementary and Alternative Medicine (NCCAM) defines complementary and alternative medicine as “a group of diverse medical and health care systems, practices and products that are not presently considered to be a part of conventional medicine. Complementary medicine is used together with conventional medicine, and alternative medicine is used in place of conventional medicine” [3]. Its integration into health care has shifted more and more to an “integrative medicine” approach which was defined by the Consortium of Academic Health Centers for Integrative Medicine as “the practice of medicine that reaffirms the importance of the relationship between practitioner and patient, focuses on the whole person, is informed by evidence, and makes use of all appropriate therapeutic approaches, healthcare professionals and disciplines to achieve optimal health and healing” [4].

In the USA, many patients now turn to interventions in complementary and integrative medicine to treat their illnesses. High usage of complementary and integrative medicine interventions, especially for patients suffering from chronic diseases, has been reported [5]. In 2007, nearly 4 out of 10 American adults had used a complementary and integrative medicine therapy in the previous 12 months [6].

As treatments become more complex (taking into account a patient's local context, subgroup membership, comorbidities, or other factors), the design of research assessing effectiveness requires flexibility in order to accommodate these various factors.

Unfortunately, many complementary and integrative medicine interventions lack the endorsement that evolves from high quality research studies; indeed, much of the evidence that supports their adoption has not made the transition from the expert level into accessible, widespread knowledge. Clearly, stakeholders (physicians, patients, payers, and others) need access to this evidence to make decisions about their treatment options. 

Nevertheless, research studies in complementary and integrative medicine are on the rise. In the USA, the National Center for Complementary and Alternative Medicine at the National Institutes of Health and other funding agencies and foundations have supported clinical, translational, and basic research on the efficacy, safety, and mechanisms of action of diverse complementary and alternative medicine modalities. However, to date, the majority of clinical trials have assessed the efficacy of medical interventions rather than their effectiveness. 

“Efficacy” refers to the extent to which a specific intervention is beneficial under ideal conditions. By contrast, “effectiveness” is a measure of the extent to which an intervention, when deployed in the field in routine circumstances, does what it is intended to do for a specific population [7]. Therefore, effectiveness can often be more relevant to policy evaluation and the health care decisions of providers and patients. Unfortunately, some efforts to achieve rigorous methodological purity have resulted in clinical results that are only marginally meaningful, because patients, interventions, and settings are not comparable to the real world. This burden presents the research community with a mandate: to discover not only efficacious treatments, but also interventions that provide the evidence critical for decisions relevant to the treatment of usual care patients. 

Drug research follows a clear hierarchical research strategy that establishes efficacy before effectiveness is evaluated. Because of its long history, complementary medicine treatments are often in widespread use before clinical research has been conducted. For complementary and integrative medicine, a reverse research strategy was recommended [8, 9]. Using a strategy that generates evidence on comparative effectiveness before determining component efficacy will help to focus on treatments that have relevance for practice and a potential for integration into health care while saving research resources. 

Because studies in Comparative Effectiveness Research (CER) are designed to be carried out in settings that reflect usual care, they have considerable potential to help health care providers as well as patients and clinicians to choose among currently available therapeutic options in complementary and integrative medicine. The Institute of Medicine defines CER as “the generation and synthesis of evidence that compares the benefits and harms of alternative methods to prevent, diagnose, treat, and monitor a clinical condition or to improve the delivery of care. The purpose of CER is to assist consumers, clinicians, purchasers, and policy makers to make informed decisions that will improve health care at both the individual and population levels” [10]. (“Alternative” does not refer to “alternative medicine” but to “best care” options.)

Among other challenges confronting the US health care system is a paucity of information about CER [11]. The current movement in conventional medicine towards more CER places strong emphasis on the evaluation of different treatment options by including more heterogeneous patients and by using less standardized treatment protocols and more patient-centered outcomes. Furthermore, stakeholder involvement is seen as highly relevant [12]. Having patients, doctors, health plan managers, hospital executives, and other stakeholders participate in the design of CER can ensure that this vital research focuses on the evidence gaps most relevant to health care decision makers [13].

CER offers a wide range of research designs and advanced techniques to distill and condense evidence from different types of studies [14] and is not limited to randomized trials but includes, among other options, the possibility of using data from observational studies or registries. Additionally, the concept of pragmatic clinical trials has emerged to describe those randomized trials that are designed explicitly to meet the needs of clinical and health policy decision-making and gain increasing acceptance by decision-makers. 

Because of the increasing and widespread use of interventions such as acupuncture, mindfulness-based interventions (yoga, meditation, etc.), and nutritional supplements to manage a variety of chronic disorders (chronic pain, cardiovascular disorders, etc.), and because there is a significant lack of evidence that supports decision-making regarding these interventions, the Institute of Medicine has identified them among its priorities for CER in complementary and integrative medicine [10].

The aim of this project was to provide recommendations for a strategic framework for CER in the field of complementary and integrative medicine.

## 2. Methods

In 2009, The Institute of Integrative Health (TIIH) and the Center for Medical Technology Policy (CMTP) cosponsored a Complementary and Integrative Medicine Stakeholder Symposium on CER. Symposium participants, including clinicians, patient advocates, payers (health insurance companies), and researchers (clinical researchers and methodologists), were selected to represent a broad range of stakeholders in the field of complementary and integrative medicine. The meeting was structured to focus attention on four central topics: (1) current evidence gaps in complementary and integrative medicine; (2) optimal study designs; (3) utilization of CER innovations from conventional medicine for complementary and integrative medicine; and (4) preferred outcomes to better inform decision-making. Experts were invited to give introductory presentations to the discussants. Meeting participants provided helpful presentations to focus conversation and drive discussion. 

From the summary of the meeting, the following recommendations for future clinical research on complementary and integrative medicine were developed and sent back to all meeting participants for comments [15]. Comments were included and the final version of the recommendations was approved by the workshop participants.

## 3. Recommendations

The following recommendations for future research in complementary and integrative medicine were developed:

**(1) Because Gaps in Evidence for Clinical and Health Policy Decision-Making Are Significant, CER Studies Should Be Made a Priority**

There is widespread and increasing use of complementary and integrative medicine, often in addition to conventional health care. Only scant evidence on comparative effectiveness of different treatment options is available and there are few data on the effectiveness of complex interventions. Because of this significant lack of data to support clinical and health policy decision-making, CER studies are urgently needed.

**(2) CER Should Engage Stakeholders at Every Stage of Research**

 Stakeholders have a vested interest in the outcomes associated with CER studies in complementary and integrative medicine. Patients, payers, and clinicians and other relevant stakeholders should be involved in every aspect of the research including identifying research priorities, study design, interpretation of results, and implementation. A mechanism is needed, through which decision-makers may communicate their needs, and that might also solicit stakeholder input for the design and implementation of CER studies. Patient input, in particular, can work to ensure that studies are likely to generate patient-relevant results.

**(3) CER Study Designs Should Highlight Effectiveness over Efficacy to Support Clinical and Health Policy Decision-Making**

Designs that emphasize effectiveness over efficacy can reshape outcomes that will be of most value to stakeholders who are faced with decision-making in usual care situations.

Such studies must (1) broaden the heterogeneity of study participants; (2) report results of subgroups in study populations even if they are exploratory; (3) ensure that research settings—including the treatment protocol—reflect usual care; (4) and also reflect usual practitioner-patient interactions.

**(4) Well-Defined Research Questions Are Prerequisites for Selecting Appropriate CER Study Designs**

Stakeholders in the field of complementary and integrative medicine embrace a vision of medicine and human health as products, procedures, pathologies, and policies enmeshed in a complex and interdependent holistic system. Consequently, the field's research questions—and its need for evidence to inform decision-making—insist that the methods used must be appropriate to answer these research questions. Although many methods are well established in CER, these methods must not determine research questions, but rather, research questions must determine methods.

A variety of study designs should be considered before deciding which is best suited for addressing the relevant research questions. The methodological advantages (and disadvantages) of (1) pragmatic clinical trials; (2) cluster randomized trials; (3) Bayesian and adaptive approaches; (4) registries and observational studies (ideally PBRN-based); and (5) other variants of design should be explored.

**(5) The Complementary and Integrative Medicine Community Should Cultivate Widely Shared Understandings, Discourse, Tools, and Technologies to Support the Use and Validity of CER Methods**

Generating support for the use and validity of methods other than RCTs requires that the complementary and integrative medicine community continue to work with others in the CER community to explore new study designs, communicate results, explore decision-making, examine evidence hierarchies, continuously share information, and evaluate its progress in meeting the goal of addressing evidentiary gaps. The responsibility for disseminating information about CER in complementary and integrative medicine should be shared among stakeholders.

**(6) Effectiveness Guidance Documents (EGDs) Should Be Developed to Shape Future CER Studies**

Driven by the information and decision-making needs of patients, payers, and clinicians and other relevant stakeholders, clear description of the details of the design of CER studies for different complementary and integrative medicine modalities is critical. In order to provide useful methodological guidance for researchers, EGDs should be developed to address such elements of study design as patient inclusion, comparators, settings, outcomes, and/or other elements. Importantly, EGDs can be strategically conceptualized to address specific clinical conditions and utilize stakeholder input to ensure that future results are applicable to real-world situations. Because CER is continuously developing, EGDs should be updated accordingly.

## 4. Discussion

These recommendations derived from a group representing different stakeholders offer many potential benefits. Among them are the development of research questions clearly linked to outcomes and relevant evidence for clinical decision-making. The development of appropriate methodological standards and guidance documents to interface with CER is associated with the framework for complex interventions [16]. Recent methodological development by the Patient Centered Outcomes Research Institute (PCORI) and the framework for complex interventions from UK can provide thorough guidance [17].

These recommendations were derived from a stepwise development process, and the active involvement of different stakeholders (clinicians, patients, payers, and researchers) ensures broader acceptability.

Although a consensus procedure was used to develop these recommendations, stakeholder participation was still limited to the group of participants. The use of more widely disseminated survey tools to increase and diversify stakeholder input could be leveraged in the future. Furthermore, opinions in these recommendations derive largely from USA-based Western perspectives and traditions. Broader participation should be solicited from more international experts, including non-Western experts and stakeholders. 

During the development process, a broader understanding of the unique methodological aspects of CER and its applicability to complementary and integrative medicine emerged. CER studies are intended to improve the external validity of clinical research to enable decision-makers to make informed decisions. Nevertheless, moving towards higher external validity simultaneously reduces the internal validity of study results. In a clinical trial, the balance between internal and external validity is not a scientific decision, but has to be carefully negotiated with the relevant stakeholder groups. Although the recommended reverse research strategy [8, 9] reflects the context and needs of complementary and integrative medicine, there is a need to discuss how contrary results on effectiveness and its components could be transferred into clinical and health policy decision-making. 

While the phenomenon of stakeholder involvement seems largely limited to CER studies in the United States at present, stakeholder participation remains a high priority. In the UK, the National Institute for Health and Clinical Excellence (NICE) has pursued a longstanding agenda prioritizing community engagement [18]. Sox (2010), reporting on the progress of CER in the US, suggested that “…all stakeholders are invited to play an active role in every aspect of CER, including priority setting, study design, and peer review” [19]. Many stakeholders have a vested interest in these recommendations. In that CER is now widely discussed in the complementary and integrative medicine community, the recommendations encourage the development of shared understandings about the terminology, methodological approaches, ethical concerns, stakeholder engagement strategies, researcher training, and the dissemination of CER research and discussion. CER is a new area for complementary and integrative medicine as it is for conventional medicine. Although, in selected areas such as acupuncture research, some contributions to CER have already been made, clear guidance is needed for future research.

The recommendations provided in this paper support the development of Effectiveness Guidance Documents (EGDs) [20]. EGDs provide detailed guidance for researchers who will pursue CER in the future. EGDs can focus on a clinical condition (e.g., osteoarthritis) or a category of clinical intervention (e.g., acupuncture) or both (acupuncture for osteoarthritis). Because the quality and breadth of available evidence varies from topic to topic, each EGD is specific and may recommend variations in study designs, which will generate the type and quality of evidence stakeholders need to support decision-making. 

## 5. Conclusion

CER is an emerging field and its development and impact have to be reflected in future research strategy within complementary and integrative medicine. This stakeholder symposium was a first step in providing systematic guidance for future CER in this field. More detailed guidance must follow.
